# “These pretzels are making me thirsty” so I’ll have water tomorrow: A partial replication and extension of adults’ induced-state episodic foresight

**DOI:** 10.1371/journal.pone.0259424

**Published:** 2021-11-17

**Authors:** Tessa R. Mazachowsky, Katarina McKenzie, Michael A. Busseri, Caitlin E. V. Mahy

**Affiliations:** 1 Department of Psychology, Brock University, St. Catharines, Canada; 2 Department of Psychology, Western University, London, Canada; University of St Andrews, UNITED KINGDOM

## Abstract

The ability to consider the future under the influence of an induced current state is known as induced-state episodic foresight. One study to date has examined adults’ induced episodic foresight and found that adults’ (like children’s) preferences for the future are related to their current state such that they predicted wanting water (vs. pretzels) in the future when experiencing a current state of thirst [1]. We attempted to replicate these findings in adults. In Study 1, adults (*N* = 198) in a laboratory selected pretzels for tomorrow at the same rate (around 20%) in an experimental condition (thirst induced) and a control condition (thirst not induced). In a lecture, 32% of adults preferred pretzels for tomorrow without thirst induction (Study 2, *N* = 63). Partially replicating Kramer et al. [1], we found that a minority of adults preferred pretzels (vs. water) when experiencing a current state of thirst. However, in contrast to their findings, our results showed that when thirst was not induced, a minority of adults also preferred pretzels for tomorrow. Thus, adults’ future preference was similar regardless of thirst induction. We also tested thirst as a mechanism for adults’ preference for the future and found that across conditions adults’ thirst predicted their choice of water (vs. pretzels) for the future. In sum, our results partially replicated Kramer et al. [1] by showing the current state, regardless of thirst induction, predicts adults’ choices for the future.

## Introduction

In daily life, humans must often consider the future and make predictions about future events, such as what clothes might be needed on an upcoming vacation, how a family reunion might transpire, or how much food you might need to purchase for the week. Because these situations have not yet occurred, humans must construct a mental representation of the future and consider what is likely to occur at a point in future time. This ability to project oneself into the future to pre-experience an event is known as episodic foresight [2,3]. Yet, anticipating how one might feel, think, or act in the future can be quite challenging [4–6], particularly when adults or children are experiencing a salient current state, such as thirst (e.g., [1]). Early evidence of this phenomenon in non-human animals, known as the Bischof-Kohler hypothesis [7], posits that some non-human animals experience difficulty anticipating future needs when not currently experiencing that need. For example, an animal that is not currently thirsty may be unlikely to anticipate future thirst. However, recent research has challenged this notion suggesting that, contrary to the Bischof-Kohler hypothesis, some non-human animals like the Western Scrub-Jay do anticipate their future needs [8–11]. In humans, Gilbert, Gill, and Wilson [12] refer to this experience as the “presentism bias” when a powerful current state interferes with an individual’s ability to predict future behaviour by way of attentional narrowing towards the present.

One study that has investigated the development of the ability to imagine one’s future self across the lifespan suggests an inverted U-shaped function where episodic foresight develops from childhood to early adulthood, peaks around 21 years of age, then declines into older adulthood [13]. Most substantial developmental improvements in episodic foresight are found to occur during early childhood between 3 and 5 years of age across a variety of tasks (e.g., [14–17]). For example, 4- and 5-year-olds were more successfully able to address psychological (e.g., avoiding boredom by selecting puzzle pieces to bring to a room containing only a puzzle board; [18]) and physiological (e.g., avoiding hunger by bringing food to a room containing no food; [19]) future needs compared to 3-year-olds. Tasks inspired by research with non-human animals, where children consider the location where an item (e.g., toys) might be needed in the future (e.g., a room without toys vs. a room with toys) have also shown improvements in the ability to make adaptive future choices in young childhood [20]. Research with young children similarly shows that, as they get older, their ability to forecast typical changes in preferences (e.g., change beverage preference as a child vs. an adult [15]) improves. Thus, children’s ability to imagine the future, select appropriate items for future use, and predict their future preferences substantially improves across childhood and reaches its peak in early adulthood. However, there is one type of episodic foresight, induced-state episodic foresight (also referred to as projection bias; see [21]), that curiously does not show this same developmental trajectory across childhood and into adulthood.

In particular, it is well documented that children struggle on induced-state episodic foresight tasks when they are asked to consider the future under the influence of a highly salient current state (e.g., thirst; [4,5,22]). One task developed to measure induced-state episodic foresight is the Pretzel task [4]. In this task, children’s baseline preference for pretzels or water is established (in a within-subjects design, only children who report a baseline preference for pretzels are included in the analysis), then thirst is induced by having them consume pretzels. After consuming pretzels and becoming thirsty, children are asked to reason about their preference of pretzels or water for tomorrow. Despite preferring pretzels over water at baseline, children experiencing thirst generally report a future preference for water (approximately 60% of the time; see [1]), presumably because of the interference from their current state. Importantly, Mahy [5] showed that thirsty children’s choice of water for the future could not be explained by “taste boredom” since children reverted to a future preference of pretzels shortly after drinking water (thus, quenching their thirst). Using the Pretzel task, children’s induced-state episodic foresight does not show any age-related improvements from early to late childhood, such that 3- to 13-year-olds similarly report a preference for having water over pretzels in the future after thirst induction [1,4,5,22–24]. These studies also show that, at the time when they make their choices for the future, children who choose water for the future are thirstier (i.e., drink more water) than children who choose pretzels (e.g., [5,24]).

We might expect performance on induced-state episodic foresight tasks to improve into adulthood, as episodic foresight abilities become more refined and practiced (e.g., [25]). Interestingly, however, the inability to overcome a current state is not unique to children, as adults also struggle to forecast future affective or physiological states. Research on adults’ affective forecasting broadly suggests that how pleased or displeased adults predict they will feel in the future often does not match their actual future emotional experience (e.g., [26]). In fact, estimating affect duration and intensity seems to be particularly challenging for adults [27]. For example, despite college student’s predictions that desirable or undesirable accommodations would affect their future happiness, one year later, student’s happiness ratings did not differ with the accommodation they were assigned [28]. Adults’ tendency to inaccurately predict the durability of their affect over time has also been shown by professors’ tendency to overestimate how long after receiving tenure their happiness would persist [27] or footballs fans’ tendency to overestimate how long their happiness would be influenced by the outcome of a game [29].

Physiological current states (e.g., hunger, thirst, exhaustion) seem to be particularly difficult for adults to overcome given the drive to reduce them, which results in attentional narrowing towards the present [30,31]. For example, Nisbett and Kanouse [6] found that hungry grocery shoppers were more likely to overestimate their food needs, resulting in them buying more food for the future than sated grocery shoppers. Similarly, Van Boven and Loewenstein [31] examined the influence of an induced physiological current state on adults’ ability to predict the physical and mental state of hikers without food or water. Results showed that adults mentioned thirst more often in their descriptions of the hiker’s feelings and predicted that the hikers would be more concerned by their thirst after vigorous exercise (inducing thirst and warmth) compared to adults who had not yet exercised [31]. Importantly, the number of adults who mentioned the hikers’ hunger did not differ with exercise, suggesting that the adults’ current state of thirst was driving their predictions for the hikers. Taken together, research with adults, similar to that with children, suggests that future forecasting is impeded by a salient current state. However, only one report has examined induced-state episodic foresight in adults and directly compared adult’s and children’s performance using the Pretzel task, which has previously only been used to measure induced-state episodic foresight in children [1].

In Study 1, Kramer et al. [1] administered the Pretzel task to 89 8-to 13-year-old children and adults to examine if children and adults’ future predictions are similarly related to their current state of thirst. In Kramer et al.’s [1] version of the task, children (*n* = 56; 2 groups: 9-year-olds and 12-year-olds) and adults (*n* = 33) were provided with 30 pretzels to eat during a 5-minute break. Participants were then asked whether they would prefer pretzels to eat or water to drink (counterbalanced) for the next day, as well as to explain their future preference choice and rate their level of thirst. Results showed that age did not predict future preference choice, that is, 17% of 9-year-olds, 31% of 12-year-olds and 21% of adults chose pretzels over water for tomorrow. Further, across age groups, 81% of participants who chose water for the future referenced thirst in their explanation for their choice. Assuming that, like children, adults generally prefer pretzels over water, such findings suggest that children and adults struggle to overcome their current state to predict their future preference. In a second study, Kramer et al. [1] sought to establish whether adults report a preference for pretzels over water without thirst induction, as is the case with children at baseline [4,5,24]. Thirty-two adults were asked to report their preference for pretzels or water for the same time tomorrow and to explain their choice after a lecture. Without inducing thirst, the majority of adults (66%) preferred pretzels for tomorrow (which given the small sample size did not differ from chance). Thus, across the two studies, Kramer and colleagues [1] found that without thirst induction, adults did not prefer pretzels to water (no different than chance performance), but following a thirst induction, adults reported a preference for water tomorrow. Overall, these results were the first to show that despite adults’ more developed cognitive abilities, they seem to experience a presentism bias and appear no better than children at forecasting the future when experiencing a salient induced current state. More generally, their findings suggest that when making decisions about the future, adults, like children, may be biased by their current state.

Our current report primarily aimed to replicate the findings of Kramer et al. [1] that showed adults struggle with induced-state episodic foresight, as is consistently found with children, and to more robustly test their conclusion that adults are influenced by their current state when making future predictions. Although Kramer and colleagues made an important contribution to the literature as the first report to examine induced-state episodic foresight in adults using the Pretzel task, their report had several shortcomings. Notably, neither of their studies used an experimental design in which participants were randomly assigned to an experimental (thirst induction) or control condition. In addition, the setting for the control condition did not match the experimental condition (i.e., the experimental thirst induction in Study 1 was conducted in a laboratory, but the control condition was conducted in Study 2 in a separate session, in a lecture). Further, both of their studies had modest sample sizes of adult participants (i.e., 33 and 32 adults, respectively), which raises questions about the precision of the estimates and the generalizability of the findings.

We sought to address such limitations of this past work by including a larger sample of undergraduate students and by using an experimental design in which participants are randomly assigned to an experimental or control condition, each conducted in the same setting. Successful replication would provide further confidence that adults indeed have difficulty forecasting the future when experiencing a physiological state of thirst just like children aged 3 to 13 years old. Furthermore, although the results from Kramer et al. [1] are suggestive of thirst as driving adults’ choice of pretzels or water for the future, this mechanism was not directly tested in either their thirst-induction (Study 1) or control conditions (Study 2) by evaluating the association between participants’ level of thirst and their choice for the future (i.e., pretzels vs. water). Overall, therefore, the present work sought to replicate and extend the findings from Kramer et al. [1] by addressing two main questions of interest: Do adults struggle with induced-state episodic foresight? Does a heightened state of thirst drive adults’ preferences for the future?

## Study 1

Following Kramer et al. [1], we had adults consume pretzels (inducing thirst; experimental condition), make a future preference choice of pretzels or water for tomorrow, provide an explanation for their choice, and rate their thirst. However, we made a few additions to strengthen their original procedure by including a large sample size of undergraduate students and a control condition where adults’ thirst was not induced. Importantly, participants were randomly assigned to either the experimental or control condition, and both conditions took place in the same laboratory context, allowing direct comparison of adults’ preferences for the future when experiencing or not experiencing a current state of thirst. Finally, we also obtained an objective measure of thirst by offering adults water to drink at the end of the study and measuring how much water they drank (following the procedure of [4]). Together, these additions allowed us to further evaluate whether, as proposed by Kramer et al. [1], adults indeed suffer impairments making choices for the future when in an induced-state of thirst. The present work also extended on their findings to allow for novel insights concerning the hypothesized mechanism underlying such impairments, that is, through directly evaluating the link between participants’ thirst (assessed subjectively using a self-report rating, objectively based on the amount of water consumed, and based on their explanations for their choice) and their choices for the future.

### Method

#### Participants

Two-hundred and sixteen undergraduate students participated (*M*_age_ = 20.92, *SD*_age_ = 4.58, range = 17–51 years; 192 females, 24 males). Participants were recruited from an Undergraduate Student Research Pool at X University. The majority of students were in their first year of study (42%) and were primarily Caucasian (71%). Eighteen participants were excluded from the final analysis for: revealing prior knowledge of the study procedure or other studies using the Pretzel task procedure with children at the end of the session (*n* = 6), failure to induce thirst in the experimental condition as indicated by lack of self-reported thirst (*n* = 2), refusal to eat pretzels (*n* = 8), hatred for pretzels (*n* = 1), or refusal to drink water (*n* = 1). All subsequent analyses were based on the remaining sample of 198 participants (*n* = 97 experimental condition, *n* = 101 control conditions; 177 females, 21 males). Note that we decided a priori to collect a sample at least three times as large (approximately 100 adults per group) as Kramer et al. ([1], *n* = 33 adults in Study 1), consistent with methodological recommendations for conducting high-power replications (e.g., [32]). Once this was achieved, we continued testing until the end of the university semester (April 2019) to allow us greater power to detect an effect half as large (i.e., φ = 0.23) as that reported by Kramer et al. ([1]; φ = 0.45). A post-hoc power analysis revealed (G*Power 3.1; [33]) that a sample size of 198 provided a very high level of statistical power to detect a medium effect between conditions (effect size w = .30, *α* = .05, power = .99; in line with a medium to large effect size, φ = 0.45, found by [1]).

#### Measures

*Induced-state episodic foresight*. We administered the same version of the Pretzel task using materials provided by the original authors [1]. However, we made two additions to their procedure: a control condition where adults were not offered any pretzels to eat and an objective measure of thirst where all adults were offered water to drink at the end of the study (a standard measure obtained in the original administration of the Pretzel task with children; [4]). Notably, these additions did not modify any of Kramer et al.’s [1] original procedures. Participants were first randomly assigned to an experimental or control condition.

**Fig 1:**
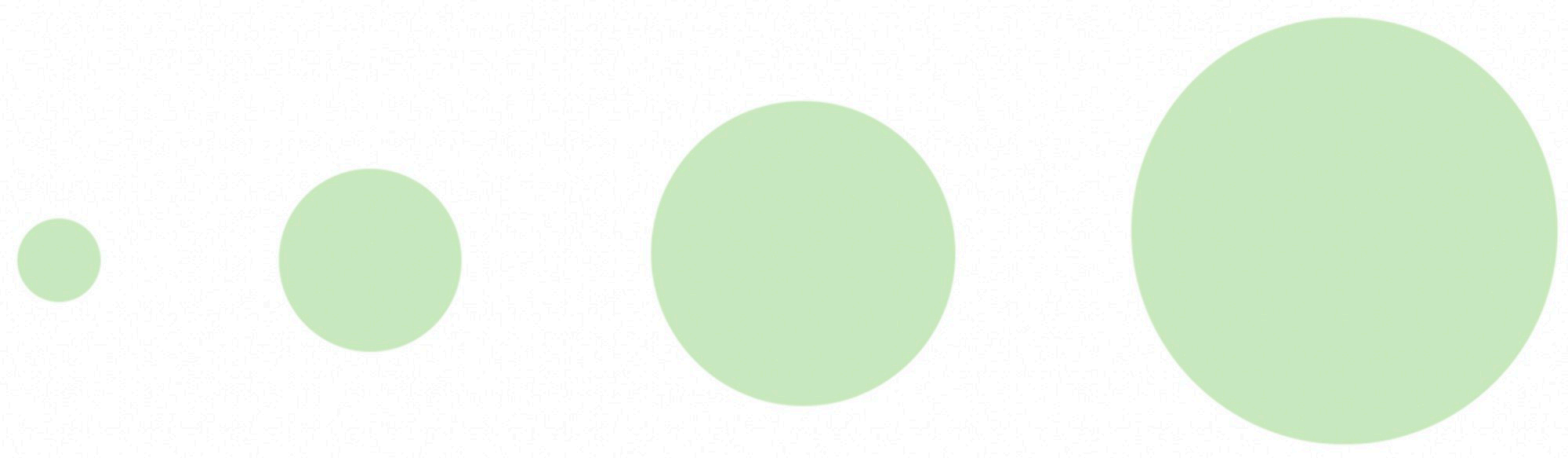
Subjective level of thirst scale.

In the experimental condition, participants provided consent and completed a short demographic questionnaire. They were then told that they were going to take a 5-minute break and encouraged to eat pretzels from a cup of 30 pretzel sticks during this time. After the 5 minutes had elapsed, the pretzels were removed, and the experimenter said: “*Let’s pretend that you’re coming here tomorrow to do some more activities and that we will take another break”*. Participants were then asked: *“What do you think you would like to have during the break tomorrow*: *some pretzels to eat or some water to drink*?*”* and simultaneously shown pictures of pretzels and water. Following their response, participants were asked to provide an explanation for their future preference choice. Participants then rated their current level of thirst (subjective thirst; from 0 = *not thirsty at all* to 3 = *very thirsty*) using a visual scale with increasingly larger circles (see Fig 1). The scale label corresponding to each circle was explained to the participants by the experimenter. Finally, the experimenter then provided participants with a 12.25 oz. water bottle and recorded the amount of water (objective thirst; 0–12.25 oz.) participants drank, along with the number of pretzels participants ate. Consistent with past administration of the Pretzel task (e.g., [4,24]), the amount of water participants drank was measured by one experimenter (who was not blind to experimental condition) using a reliable kitchen scale. In the control condition, an identical procedure was followed except participants were asked to sit quietly during the 5-minute break instead of being offered pretzels. The experimenters were not blind to condition, since the condition was revealed when pretzels were provided to participants; however, the experimenters were not privy to the overall goal of the study.

In both conditions, the order in which the response options (pretzels and water) were presented during the question about what they would like to have the next day was counterbalanced (98 participants were asked if they would like pretzels first then water, and 100 participants asked if they would like water first then pretzels). After the study was completed, participants were asked to report whether they had any prior knowledge of the study and were debriefed and invited to ask questions about the study at that time. Participants received course credit for their participation. Data collection began in Spring 2018 (only 11 participants were collected during this time due to low enrollment during Spring term at the institution) and continued over the course of two university semesters (September 2018 to April 2019). All procedures were approved by the research ethics board at X University (*Adults’ future preferences* [file number: 17-342-X]).

Using the coding scheme provided by Kramer et al. [1], we coded participants’ explanations for the presence of implicit and explicit thirst. Participants’ explanations were coded as demonstrating *explicit* thirst if their explanations referenced *thirsty* or *parched*. Participants’ explanations were coded as demonstrating *implicit* thirst if their explanations referenced *dry*, *hydrating*, *refreshing*, *hot outside*, or *salty*. Explanations that did not include any implicit or explicit thirst-related terms were coded as not referencing thirst (see Table 1). Agreement between two independent coders across all explanations was almost perfect (κ = .98).

**Table 1:** Future preference choice explanation examples by coding category.

Coding Category	Example Explanation
Explicit thirst	“Just cause I just finished the pretzels so I’m thirsty
	“Kind of parched”
Implicit thirst	“Because pretzels made my mouth dry”
	“Because the pretzels are salty and I feel like I need a little water right now”
Unrelated to thirst	“I don’t often eat pretzels but I always drink lots of water”
	“I don’t know, it’s easier, cleaner”

### Results and discussion

#### Preliminary analyses

Participants’ future preference choice was not related to: sex, *χ*^*2*^(1) = 2.51, *p* = .11; total pretzels consumed (*M* = 17.69 pretzels, *SD* = 9.78), *Wald* (1) = 0.74, Exp (*B*) = 1.02, *p* = .39; ethnicity (Caucasian versus not), *χ*^*2*^(1) = .001, *p* = .98; or time since their last meal (*M* = 7.38 hours, *SD* = 6.37), *Wald*(1) = 0.26, Exp (*B*) = 1.01, *p* = .61. These factors were not included in further analyses. Presentation order of pretzels or water was related to future preference choice, *χ*^*2*^(1) = 4.82, *p* = .03, φ = -.16, such that participants chose water more frequently when it was presented first (86% vs. 73.5% chose water when it was presented second) and pretzels more frequently when it was presented first (26.5% vs. 14% chose pretzels when it was presented second). This factor was thus examined further in the main analysis, as detailed below.

#### Main analyses

**Fig 2:**
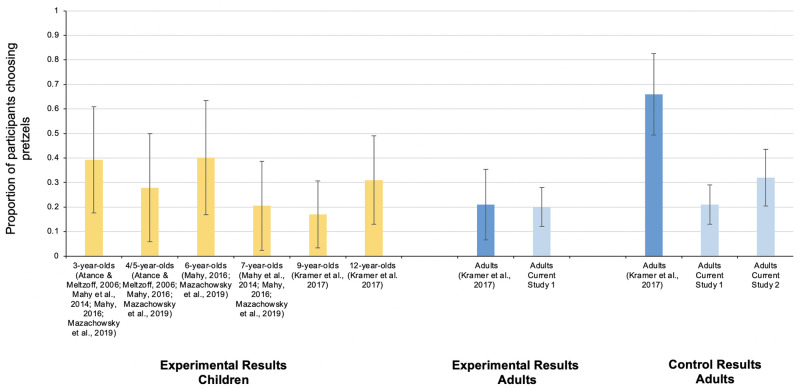
Pretzel task performance across age using previously collected data and the current study. Error bars represent 95% confidence intervals using weighted means.

*Future preference choices*. Proportion of pretzels versus water choices did not differ by condition, *χ*^*2*^(1) = .05, *p* = .83, φ = -.02. In the control condition, 21% of participants chose pretzels for tomorrow, while 20% of participants in the experimental condition chose pretzels for tomorrow (see Fig 2 for confidence intervals). Results were similar when presentation order was included as a moderating factor: In a logistic regression model predicting future preference choice from condition, order of presentation, and their interaction, each predictive effect failed to reach significance. That is, the proportion of pretzels versus water choices did not differ between conditions as a function of presentation order: pretzel option presented first, *χ*^*2*^(1) = .05, *p* = .83, φ = -.02; water option presented first, *χ*^*2*^(1) = .00, *p* = 1.00, φ = .00). Thus, regardless of condition, a minority of participants (20%) reported a preference for pretzels over water in the future and this was significantly below chance, *χ*^*2*^(1) = 70.32, *p* < .001.

Notably, we found the same pattern of results in our experimental condition as Kramer et al. (2017, Study 1) who also found that a minority of adults (21%) preferred pretzels over water for tomorrow in their thirst induction condition. However, unlike Kramer et al. [1], who found that a majority of participants (66%) preferred pretzels for tomorrow in their control condition (Study 2), only a minority of adults in our control condition also preferred pretzels over water for tomorrow. Consequently, we cannot conclude based on their choices for tomorrow that adults struggled with induced-state episodic foresight since they preferred water (over pretzels) for tomorrow regardless of whether they experienced a thirst induction.

*Subjective and objective indicators of thirst*. Subjective and objective indicators of thirst were significantly positively related, *r*(196) = .29, *p* < .001. A multivariate ANOVA revealed a significant multivariate effect of condition on participants’ thirst, *F*(2,195) = 4.85, *p* = .009, η^2^ = .05. With respect to the individual thirst indicators, subjective thirst did not differ significantly by condition, *t*(196) = -1.83, *p* = .07. However, participants in the experimental condition subjectively reported a (non-significantly) higher level of thirst (*M* = 1.93, *SD* = 0.73) than participants in the control condition (*M* = 1.73, *SD* = 0.77). Furthermore, the amount of water adults drank (our objective measure of thirst) significantly differed by condition, *t*(196) = -2.93, *p* = .004. Participants in the experimental condition drank significantly more water (*M* = 3.57 oz, *SD* = 2.88) than participants in the control condition (*M* = 2.46 oz, *SD* = 2.44).

Next, using a logistic regression, we examined if thirst predicted adults’ future preference choice across all participants, separately for objective and subjective thirst indicators. Objective thirst significantly predicted adults’ future preference choice, *Wald* χ^2^(1) = 6.60, *p* = .01, Exp(*B*) = 0.52 (see S1 Appendix for logistic regression tables). Thus, on average, for every one standard deviation unit increase in objective thirst, the odds of choosing pretzels decreased by 48%. A follow-up analysis indicated that this pattern overall did not differ significantly across conditions, as revealed by a non-significant interaction between condition and objective thirst in predicting future preference choice (effect of objective thirst in experimental condition, *Wald* χ^2^(1) = 2.74, *p* = .10, Exp(*B*) = 0.82; effect of objective thirst in control condition, *Wald* χ^2^(1) = 4.24, *p* = .04, Exp(*B*) = 0.72).

Similarly, subjective thirst significantly predicted adults’ future preference choice, *Wald* χ^2^(1) = 17.83, *p* < .001, Exp(*B*) = 0.41. On average, for every one standard deviation unit increase in subjective thirst, the odds of choosing pretzels decreased by 59%. This pattern was consistent across conditions, as revealed by a non-significant interaction between condition and subjective thirst in predicting future preference choice (effect of subjective thirst in experimental condition, *Wald* χ^2^(1) = 6.73, *p* = .009, Exp(*B*) = 0.34; effect of subjective thirst in control condition, *Wald* χ^2^(1) = 11.22, *p* = .001, Exp(*B*) = 0.27).

Together, these findings show that, as Kramer et al. [1] suggested, adults’ physiological state of thirst, both objective and subjective thirst, was driving their future preference choice. Such results may be consistent with a presentism bias [12]. Notably, however, because participants experiencing greater thirst tended to choose water over pretzels for tomorrow regardless of whether they were subject to a thirst induction, we cannot conclude that adults in the experimental condition were necessarily inaccurate in forecasting the future when experiencing a current state of thirst. Consequently, we cannot conclude based on these findings that adults struggle with induced-state episodic foresight.

*Explanations for adults’ future preference choice*. Overall, 59% of participants’ explanations referenced thirst (implicit or explicit). Furthermore, participants’ explanations contained references to being thirsty more frequently in the experimental than control condition, χ^2^(1) = 4.28, *p* = .04, φ = .15, such that, in the experimental condition, 66% of participants’ explanations referenced thirst (implicit and explicit), whereas in our control condition, 52% of participants’ explanations referenced thirst. A binomial test indicated that in the experimental condition, participants’ reference to thirst (66%) was significantly above chance (*p* = .002), whereas participants’ reference to thirst in the control condition (52%) was at chance, *p* = .84). Such findings provide further evidence that our experimental manipulation heightened adults’ level of thirst.

Across all participants, adults’ explanations significantly predicted their future preference choice, *Wald* χ^2^(1) = 29.09, *p* < .001, Exp(*B*) = 0.08. More specifically, of the adults mentioning thirst in their explanations, 5% chose pretzels; in comparison, among those not mentioning thirst in their explanations, 42% chose pretzels. A follow-up analysis indicated that this pattern was consistent across conditions, as revealed by a non-significant interaction between condition and explanations in predicting future preference choice (effect of explanation in experimental condition, *Wald* χ^2^(1) = 16.40, *p* < .001, Exp(*B*) = 0.08; effect of explanation in control condition, *Wald* χ^2^(1) = 12.59, *p* < .001, Exp(*B*) = 0.06). More specifically, in the experimental condition, among adults mentioning thirst in their explanations, 6% chose pretzels, in comparison to 46% of those not mentioning thirst in their explanations. Similarly, in the control condition, among adults mentioning thirst in their explanations, 4% chose pretzels, in comparison to 39% of those not mentioning thirst in their explanations.

Also, among adults in the experimental condition whose future preference was water, 77% referenced thirst (45% explicitly and 32% implicitly), while only 21% of participants choosing pretzels referenced thirst. Of the participants in the control condition who chose water, 63% referenced thirst (38% explicitly and 25% implicitly), while only 10% of participants choosing pretzels referenced thirst.

Overall, these findings provide additional evidence that in both conditions, adults’ thirst was driving their future preference choice. Moreover, regardless of whether thirst was experimentally induced, adults’ predictions for whether they would prefer pretzels or water for the future were guided by their current level of thirst, as reflected in how much water they drank, their self-reported thirst, and by their references to thirst in their explanations.

## Study 2

In Study 1, the thirst induction manipulation did not impact participants’ future preference for pretzels (over water). Rather, the minority of participants in both the experimental and control conditions preferred pretzels over water for tomorrow. Of note, findings from our experimental condition in Study 1 are consistent with some of the results from Kramer et al. ([1], Study 1) in that only a minority of participants chose pretzels (over water) for tomorrow following a thirst induction (20% in our Study 1, 21% in their Study 1). However, in our control condition only 21% of participants reported preferring pretzels for tomorrow (see Fig 2), which failed to replicate Kramer et al.’s ([1], Study 2) finding that 66% of participants (at chance), who did not undergo a thirst induction, preferred pretzels for tomorrow.

Notably, our manipulation did significantly increase participants’ thirst, as revealed by the amount of water they consumed, their self-reported thirst (at a trend level), and their explanations. Furthermore, participants’ thirst predicted their future preference for water over pretzels, and this link between heightened thirst and future preference for water (vs. pretzels) was found in both conditions. Overall, in line with Kramer et al. [1], our Study 1 results suggest adults’ preferences for the future were tied to their current thirst.

In summary, although we did not find evidence that the thirst induction manipulation influenced adults’ episodic foresight as proposed by Kramer et al. [1], we did find evidence that (regardless of experimental condition) adults’ preferences for the future were tied to their current state of thirst. One of the reasons for these contradictory findings could be a result of the design in Kramer et al. [1] in which participants in Study 2 indicated their preference (when thirst was not induced) in a different environment (a lecture) than the experimental condition in Study 1 (a laboratory). In contrast, in Study 1 of the current report, participants were randomly assigned to an experimental or control condition, and all participants made their future preference choice in the same controlled laboratory environment. Thus, in Study 2, we sought to investigate whether asking students to report their preference (i.e., without inducing thirst) for pretzels or water in a lecture setting (similar to Kramer et al.’s [1] Study 2) would produce different results than in a laboratory setting.

### Method

#### Participants

Seventy-four undergraduate students participated (*M*_age_ = 21.14, *SD*_age_ = 5.07, range = 18–45 years; 51 females, 18 males, 5 students did not complete demographics). Participants were recruited from three second-year undergraduate psychology lectures at X University (13 students were from a Spring late-evening lecture, 30 students were from a Fall afternoon lecture, and 31 students were from a Fall evening lecture). The majority of students were in their second year of study (68%) and were primarily Caucasian (72%). Eleven participants were excluded from the final analysis for indicating in the questionnaire package that they had previously heard about the study or the Pretzel task procedure with children. We used this question to screen participants who may have previously participated in Study 1; however, it is possible that participants from Study 1 could have participated in Study 2. No participants reported an explicit hatred for pretzels in their explanations. All subsequent analyses were based on the remaining 63 participants (43 females, 16 males, 4 students did not complete demographics). A post-hoc power analysis revealed (G*Power 3.1; [33]) that a total sample size of 165 (i.e., 101 control condition participants in Study 1 vs. 64 in Study 2) provided a very high level of statistical power to detect a medium effect between Study 2 and the control condition in Study 1 (effect size w = .30, α = .05, power = .97).

#### Measures and procedure

At the end of three separate undergraduate psychology lectures, students were invited to participate in an optional after class activity. Interested students were provided with a package in a stapled, set order that included: (1) a consent form (on which participants were only told they would make choices about their preferences for the future and did not reveal the purpose of the study; see S2 Appendix), (2) one future preference question (asking participants to choose between pretzels or water and then explain their choice), (3) two additional questions asking about the time since participants’ last meal, and prior knowledge of the study (i.e., “Had you heard anything about the study prior to today? If yes, explain briefly.”), and (4) demographic questions (e.g., age, sex, academic major, year of study, overall average, and ethnicity), which were collected to ensure similar sample characteristics as Study 1. Critically, questions were completed in the fixed order above and were placed on separate pages so that *only* the consent form was completed prior to the future preference question as required by the University Research Ethics Board. All other questions (i.e., time since last meal, prior knowledge of study, and demographics) were completed after the participant made their future preference choice and thus could not have influenced their response. No other questions were asked of participants. On the future preferences questionnaire, students were presented with a picture of pretzels and water (with corresponding written labels below) and were asked to circle “What they would like to have at this same time tomorrow?”. Participants were also asked to provide a written rationale for their choice. Data for Study 2 was collected in May and November 2019. All procedures were approved by the research ethics board at X University (*Adults’ future preferences* [file number: 17-342-X]).

### Results and discussion

#### Preliminary analyses

Participants’ future preference choice was not related to: sex, *χ*^*2*^(1) = 1.34, *p* = .25; ethnicity (Caucasian versus not), *χ*^*2*^(1) = .28, *p* = .60; or time since their last meal (*M* = 5.86 hours, *SD* = 5.23), *Wald*(1) = 0.13, Exp (*B*) = 1.02, *p* = .72. These factors were not included in further analyses.

#### Main analyses

*Future preference choices*. Across lectures, 32% of adults reported a preference for pretzels over water for the next day, suggesting that when thirst is not induced the majority of adults prefer water for the next day (see Fig 2 for confidence intervals). This percentage was significantly below chance, χ^2^(1) = 8.40, *p* = .004. The proportion of adults choosing pretzels over water did not differ across lectures, (*Fisher’s Exact Test* = 2.27, *p* = .36). Thus, our results suggest that in an undergraduate lecture setting, the majority of participants had a preference for water (vs. pretzels) for the next day. Such findings are consistent with our results from Study 1. Indeed, across our two studies, the proportion of adults reporting a preference for pretzels over water in our Study 1 control condition (21%), did not significantly differ from the proportion of adults reporting a preference for pretzels over water (32%) in Study 2, χ^2^(1) = 2.48, *p* = .12, φ = .12. However, our results do not align with Kramer et al. ([1], Study 2), who found that 66% (not significantly greater than chance) of students in an undergraduate lecture reported preferring pretzels over water for tomorrow.

*Explanations for adults’ future preference choice*. Overall, 24% of participants’ explanations referenced thirst (implicit or explicit). Participants’ explanations were significantly related to their future preference choice, χ^2^(1) = 9.42, *p* = .002, φ = -.39. Notably, all of the adults (100%) mentioning thirst in their explanations chose water for the future, as did 57% of those who did not mention thirst in their explanations. Adults’ explanations also revealed that 36% of participants who chose water referred to thirst (10% explicitly referenced thirst and 26% implicitly reference thirst). Of the participants who chose pretzels, 100% of explanations were unrelated to thirst. Overall, compared to our control condition in Study 1, participants in Study 2 referenced thirst less frequently (24% in Study 2 versus 52% in Study 1) in their explanations, χ^2^(1) = 11.82, *p* = .001, φ = .27. Such findings suggest adult participants were not experiencing a high level of thirst in the lecture setting, in general, when reporting their preference for tomorrow. Nonetheless, in support of Kramer et al. [1], our results provide further evidence that, even outside of a laboratory context, adults’ preferences for tomorrow are linked with their current state of thirst.

## General discussion

The current work examined if, as reported by Kramer et al. [1], adults experience difficulty with induced-state episodic foresight, and whether experiencing a salient current state of thirst biases adults’ ability to reason for the future. Extending on their work, we sought to directly evaluate whether adults’ current thirst drove their preferences for pretzels or water in the future using the Pretzel task. In Study 1, we found that, when thirst was induced, the majority of adults chose water over pretzels for the next day. However, the proportion of adults choosing water in the experimental condition did not significantly differ from the proportion of adults choosing water in the control condition, when thirst was not induced. Further, regardless of condition, adults’ thirst (subjective thirst, objective thirst, and open-ended explanations) predicted their future preference choice, suggesting that participants’ current state of thirst was influencing their reasoning about the future. In Study 2, when thirst was not induced, we again found that the majority of adults surveyed in a lecture setting chose water over pretzels for the next day, mirroring the results of the experimental and control conditions in Study 1. Furthermore, in Study 2 participants’ thirst, as conveyed by their explanations, predicted their choice for the future. Thus, across both studies, regardless of our thirst manipulation, most adults chose water for tomorrow and thirst appeared to drive their preferences for the future.

The first goal of the current study was to replicate previous findings by Kramer et al. [1] that suggested adults struggle with induced-state episodic foresight. Replicating Kramer et al. [1], we found that when thirst was induced, a minority of adults chose pretzels for the next day (20% in our experimental condition in Study 1 vs. 21% in Kramer et al. [1], Study 1). By itself, this result is consistent with their suggestion that adults struggle with induced-state episodic foresight; however, our control condition results make it difficult to determine whether this is the case given that adults in our studies preferred water over pretzels even without thirst induction. Indeed, we failed to replicate Kramer et al.’s ([1], Study 2) results that almost two-thirds of adults prefer pretzels over water when thirst was not induced (although this proportion was no greater than chance). In both our control condition in the laboratory (Study 1) and a lecture setting (Study 2), we found that a minority of adults’ preferred pretzels over water for tomorrow (Study 1: 21%; Study 2: 32%), whereas Kramer et al. [1] found a majority of adults (66%, Study 2) preferred pretzels over water for tomorrow in their control condition in a lecture setting. Taken together, our results suggest that adults prefer water over pretzels regardless of the manipulation of their current state of thirst, and regardless of the setting (lab or lecture), which raises questions about the suitability of the Pretzel task for use with adults given that our findings suggest that adults generally do not seem to prefer pretzels.

There are several possible reasons why we did not fully replicate Kramer et al.’s [1] results. First, Kramer et al.’s [1] control condition in a lecture setting lacked the same stringent control as a laboratory setting. Drawing on participants’ explanations from the current report, participants in lecture (Study 2) reported less thirst than participants in lab (Study 1), which suggests the importance of an appropriately controlled context where water intake during the study can be restricted. Thus, it is not possible to know whether the difference that Kramer et al. [1] report in adults’ preferences when thirst was induced (their Study 1) versus not induced (their Study 2), was due to the thirst induction or the environment in which they made their preference choice, as the two were confounded in their work. To address this issue, we had adults report their future preference choice when thirst was not induced in a laboratory (our Study 1) and in a lecture (our Study 2). We found similar results in both of these settings (i.e., adults preferred water for the future). However, our results in Study 2, when adults made a future preference choice at the end of a lecture, failed to replicate Kramer et al.’s [1] results in a similar setting. Notably, because our experimental and control conditions in Study 1 were both conducted in the same lab, we can be confident that our results are not due to a difference in environment.

Second, the time of day Kramer et al. [1] surveyed adults in lecture (this information was not provided in their report) could have influenced their level of hunger, resulting in them being more likely to choose pretzels (vs. water), which could explain why we did not replicate their findings. Alternatively, participants in our current Study 2 could have been sampled on particularly hot or dry days, accounting for a higher proportion of our participants choosing water over pretzels. To address this issue, we sampled adults for Study 2 from multiple lectures in different seasons (early spring and fall) and different times of day (evening and afternoon) to avoid the possibility that choice was a result of a specific time of day or the weather during a particular season. Importantly, we found no significant differences in future preference choice as a function of season or time of day.

Third, it may be the case that there has been a shift in adults’ tendency to make health-conscious choices, or their desire to be perceived as health conscious since Kramer et al.’s collection of data in 2014 (vs. 2019 for the present studies). A shift in adults’ desire to be healthy could explain why the majority of adults in our study chose water over pretzels. However, we found that overall, only a small number of adults (four adults in the control condition and two in the experimental condition in Study 1; one adult in Study 2) explained their choice for water for health-related reasons (i.e., used the word *healthy* or *health* in their explanation; e.g., “I drink water consistently throughout the day; I am a fairly healthy person”). Thus, health consciousness does not seem to explain why the majority of adults in both of our studies chose water (vs. pretzels) even when thirst was not induced.

Fourth, there may have been differences in our undergraduate adult sample’s demographic characteristics (e.g., socioeconomic status, race/ethnicity) compared to Kramer et al.’s [1] that could have led to different rates of adults selecting pretzels over water. For example, our sample was predominately Caucasian (> 70%), while Kramer et al.’s [1] adult sample was more diverse (i.e., 18% Caucasian, 18% Hispanic, 42% Asian, 21% percent other). Research has shown that socioeconomic status influences adults’ decision making, such as placing greater value on immediate needs (e.g., see [34] for review).

Finally, differences in pretzel preference between Kramer et al.’s [1] Study 2 and the current Study 2 could have arisen from our administration of a consent form. Unlike participants in Kramer et al.’s [1] second study, our Study 2 participants were required to complete a consent form prior to participation in accordance with standard ethical guidelines, which could have resulted in different rates of adults’ pretzel preference between studies. Although, it seems unlikely that the consent process would have biased participant responses, given that the purpose of the study was vaguely described in the consent form (i.e., it was described that participants would be asked to make a future preference choice).

Notwithstanding these potential explanations for the differences between our findings and theirs, our partial replication of Kramer et al.’s [1] finding concerning adults’ difficulty with induced-state episodic foresight has implications for the understanding of the development of induced-state episodic foresight across childhood and into adulthood. Previous research has consistently found an absence of developmental progression in children’s induced-state episodic foresight ability [1,4,5,22]. From 3 to 13 years old, children typically perform poorly on the Pretzel task and report a preference for water over pretzels in the future following thirst induction, despite reporting a preference for pretzels at baseline [5,22]. These findings support two theoretical perspectives: (1) the Bischof- Kohler hypothesis, which suggests that future motivational states, such as thirst, may be difficult to anticipate if that state is not currently being experienced [7]; and (2) the presentism bias, which suggests that future decisions may be tied to one’s current state [12].

Yet, despite children’s poor performance on the Pretzel task, there is evidence that they might be increasingly aware that thirst is driving their future preference choice (e.g., [1,5]), which aligns with developmental improvements in metacognitive awareness across childhood (e.g., [35]). Interestingly, the lack of development in episodic foresight is unique to tasks like the Pretzel task in which children’s current physiological state is induced. When children’s current physiological state is not manipulated, children’s episodic foresight performance improves substantially across childhood (e.g., [4,15]).

It is less clear, however, whether induced-state episodic foresight fails to develop across childhood and reach its peak in adulthood. Adults’ difficulty predicting future affective and physiological states has been supported by research showing that adults: mispredict how long their happiness will endure after an event (e.g., football game win; [29]), overestimate future food needs when hungry (e.g., [6]), or project their current physiological state onto others, resulting in poor predictions [31]. Most recently, Kramer et al. [1] reported similar induced-state episodic foresight performance in adulthood as in childhood using the Pretzel task. However, adults in both of our studies appeared to prefer water over pretzels (and regardless of thirst induction), so we cannot be certain whether this choice represents difficulty, inaccuracy, bias, or a rational choice in predicting their preferences for the future.

Indeed, consistent with a developmental perspective on meta-cognitive awareness [35], it is possible that adults use their current state to anticipate their future preferences for a similar situation (in this case, a similar situation arising the next day). From this perspective, one’s current state (thirst) may facilitate, rather than impede or interfere with, an optimal choice for the future. This idea is supported by the insurance hypothesis, which proposes an evolutionary adaptive strategy in which a currently food-insecure environment cues a physiological reaction of one’s body to store fat (e.g., by reducing energy expenditure), thereby ensuring survival and reproduction in the future if resources are scarce. Although in contemporary environments this strategy can be problematic and lead to obesity [36], from an evolutionary standpoint it is adaptive because it assumes similar resource availability between current and future environments and, thus, one’s physiology anticipates future food scarcity in line with the current circumstances. Conversely, research examining individual differences in relation to adults’ future choices finds associations between adults who chose pretzels over water for the future and better working memory and higher college grade point averages (e.g., [1]). Additional research is thus needed to further evaluate these various notions concerning the accuracy and bias in adults’ preferences for the future.

With respect to the second main goal of the present work, findings from both of our studies—spanning experimental and control conditions, and laboratory and lecture settings—support the general conclusion of Kramer et al. [1] and other findings with children (e.g., [4,5]), that one’s current state of thirst (particularly as reflected in their explanations and their objective thirst, as found in our study) is linked with their future predictions. Even though the majority of our adult participants preferred water over pretzels regardless of condition or setting, the choice of water for tomorrow was stronger among adults indicating that they were thirstier. Such findings may be consistent with the presentism bias [12], according to which adults’ current state of thirst interfered with their predictions for the future; however, to provide evidence that such a bias exists in adults on the Pretzel task, it would need to be demonstrated in a future study that adult’s actual choice the following day differed from their original prediction (i.e., a preference reversal).

Importantly, in support of proposals by Kramer et al. [1], our results provide direct evidence of a link between future preference for water (vs. pretzels) and current thirst, based on several types of indicators, most notably an objective measure of thirst (the amount of water they drank; Study 1), and open-ended explanations for future preferences (Study 1, Study 2). Further, whereas previous studies examining episodic foresight using the Pretzel task have speculated about thirst as the mechanism underlying children’s and adults’ future preferences [4,5,24], the present work provides direct evidence of this by showing a robust statistical link between heightened current thirst and greater likelihood of preferring water (over pretzels) for tomorrow. Specifically, we found that regardless of condition, adults’ thirst, particularly their objective thirst and their explanations for the choice, predicted their future preference choice for water. Thus, our findings generally support the conclusion from Kramer et al. [1] that adults’ preferences for the future are linked with their current state. Our studies also expand on their work with a more robust and direct test of this phenomena, providing a novel contribution to understanding the developmental trajectory of induced-state episodic foresight. Indeed, our findings broadly support the idea that, even in early adulthood, induced-state episodic foresight is tied to an individuals’ current state, and thus young adults might not be immune to the impact of the current state on their future thinking (e.g., [6]).

Extending on Kramer et al.’s [1] past work, yet remaining consistent with their procedure and administration of the Pretzel task, we attempted a more vigorous test of the influence of the current state on future reasoning with several additions: a larger sample size, random assignment to a control condition, and an objective measure of thirst. Based on our findings, we propose further recommendations for the future administration of the Pretzel task with adults, in order to ensure a stronger manipulation of the current state.

First, future studies should consider determining adults’ baseline preference for pretzels or water to ensure participants’ future preference choice after thirst induction is a result of the manipulation. With a sufficiently large sample size and adequate experimental control, the use of random assignment should ensure that results from the control condition provide a valid proxy for what would have been observed at baseline in the experimental condition. Nonetheless, this reasoning based on random assignment to a control condition assumes similar distribution of participants across groups that initially preferred water over pretzels and had varying levels of both state and trait thirst.

An alternative approach would be to ask adults to report their current preference *before* thirst is induced, so that participants who report a baseline preference for water over pretzels could be removed from subsequent analysis. If adults generally prefer water over pretzels, as the results of the current study suggest, then this may suggest the Pretzel task is not suitable for use with this age group. Research using this approach with children (e.g., [5,24]) consistently reports that the majority of children prefer pretzels (versus water) when asked at baseline. Thus, it is especially important for future work to include baseline measures of adults’ preferences to be confident adults also prefer pretzels prior to thirst induction, as well as to determine if they can overcome their current state when reasoning for the future. Measuring adults’ baseline preference would also allow for the examination of adult’s preference reversal. With child samples, Mahy [5] showed that majority of children selected pretzels at baseline, but choose water after thirst induction, then reverted to their baseline preference of pretzels after their thirst was quenched. However, having adults explicitly state their preference at baseline may lead them to maintain consistency in their response even after thirst is manipulated. Lending support for this idea, Read and van Leeuwen [37] found that adults who predicted they would choose a healthy snack one week prior, were more likely to maintain consistency with this prediction when offered a healthy or unhealthy snack again in the present, even if they were hungry at the time of consumption.

Second, future studies would also do well to include additional experimental conditions where adults make a preference choice for the present after thirst is induced to better determine the effect of the current state on present versus future reasoning (e.g., [4]), or make choices between water and a food item that differs from the food provided to induce thirst (i.e., pretzels) to rule out sensory-specific satiety (for review see [38]). For example, research has shown that when preschool children who have consumed pretzels to induce thirst are given the choice between water and a novel, more motivating food item (i.e., a cupcake), the majority of children choose a cupcake over water for both the future and the present [39]. Thus, the influence of the current state on preference choice may be dampened when a novel food item is presented.

Third, future studies may consider measuring objective thirst using two independent raters to establish interrater reliability when recording the ounces of water drank and ensure that these raters are blind to condition. Although in the current study we found our objective and subjective indicators of thirst were related, suggesting consistency among thirst indicators.

Fourth, the current study consisted of more female than male participants. Although future preference choice did not differ with sex, the imbalance in females to males in our sample may limit the generalizability of our findings.

Fifth, the wording of the future preference choice question (i.e.,*“What do you think you would like to have during the break tomorrow*: *some pretzels to eat or some water to drink*?*”*) is hypothetical, such that adults “pretend” they will return the next day, but do not actually do so, which may limit the ecological validity of the task. Some past work with adults has provided them with real choices (e.g., choice of snacks they would like to consume one week in the future) and then measured their actual choice in the future at the time of consumption [37]. However, measuring adult’s real choices has yet to be examined using the Pretzel task. It is also possible that the wording “let’s pretend” may have encouraged psychological distancing. For example, Trope and Liberman [40] have suggested hypotheticality along with temporal, spatial, and social distance are important dimensions of psychological distance. Importantly, psychological distancing has been found to improve adult’s reasoning (e.g., [41]). We might expect, then, that adults would experience less difficulty overcoming their current state to reason for the future if engaging in psychological distancing (as has been found with children using the Pretzel task [24]), which does not appear to be the case in the present studies given that participants’ current thirst predicted their choice for water the next day. Thus, future work may seek to adapt the Pretzel task so adults’ preferences are directly (rather than hypothetically) evaluated.

Finally, future studies should consider quenching adults’ pre-existing hunger or thirst before the task (e.g., apple juice provided to children before the Pretzel task; [5]). Ensuring control participants are not thirsty (or hungry) is particularly important since it is typically assumed that this group is not influenced by a current state when making their future preference choice. On this point, we note our results from the control condition in Study 1 demonstrated that even without a thirst induction, adults’ preferences for the future were linked with their current thirst. Further, it is clear from participants’ explanations in our control condition in Study 1 that some adults were thirsty upon arrival into the lab. For example, 63% of adults in the control condition explained their choice for water by referencing thirst. However, inferring thirst through adult’s explanations should be interpreted with caution since it is possible that participants could have been thirsty, but did not mention thirst in their explanations. Together, these findings suggest thirst was motivating participants’ future preference choice for water, even in the control condition.

Overall, our results provide both a partial replication and an extension of the findings reported by Kramer et al. [1]. That is, we found support for Kramer et al.’s [1] conclusion and other work using the Pretzel task with children (e.g., [4,5]), that humans may be influenced by their current state. However, we did not replicate the finding that adults report no preference between pretzels and water when thirst was not induced. Instead, using random assignment and a careful control condition that matched our experimental condition, we found that adults reported a future preference for water—and that such a preference was linked with their thirst–*regardless* of whether thirst was induced. Thus, our results support Kramer et al.’s [1] overall assertion that adults’ forecast for the future is tied to their current state, but also highlight the need for direct evidence concerning whether or not induced physiological states such as thirst interfere with adults’ ability to accurately forecast their preferences for the future, as is consistently reported with children. It is important to know if induced-state episodic foresight improves across the lifespan since poor forecasting can have dire consequences (e.g., [30]). If this ability does not improve across the lifespan, it could be targeted for intervention. More broadly, future work is needed to determine the degree to which adults’ preference for water (vs. pretzels) for the future reflects the impact of their current state of thirst on future reasoning versus a general preference for water.

## Supporting information

S1 Appendix Logistic regression tables. (PDF)

S2 Appendix Study 2 consent form. (PDF)
