# Supplementary material for: “These pretzels are making me thirsty” so I’ll have water tomorrow: A partial replication and extension of adults’ induced-state episodic foresight
Source: PLoS One. 2021 Nov 17;16(11):e0259424. doi: 10.1371/journal.pone.0259424 (PMC8598010; doi:10.1371/journal.pone.0259424)
Supplement: S1 Appendix — (PDF) [file pone.0259424.s001.pdf]

S1 Appendix: Logistic Regression Tables

Study 1 logistic regression analyses

Objective and subjective thirst across conditions

Table 1

Logistic regression analysis of objective thirst predicting future preference choice across conditions

Predictor	*B* (SE)	*Wald* χ^2	Exp(*B*)	95% CI
Objective thirst	-.66 (.26)	6.60	.52	.31 - .86

Table 2

Logistic regression analysis of subjective thirst predicting future preference choice across conditions

Predictor	*B* (SE)	*Wald* χ^2	Exp(*B*)	95% CI
Subjective thirst	-.89 (.21)	17.83	.41	.27 - .62

Objective thirst between conditions

Table 3

Logistic regression analysis of objective thirst predicting future preference choice in the experimental condition

Predictor	*B* (SE)	*Wald* χ^2	Exp(*B*)	95% CI
Objective thirst	-.20 (.12)	2.74	.82	.65 – 1.04

Table 4

Logistic regression analysis of objective thirst predicting future preference choice in the control condition

Predictor	*B* (SE)	*Wald* χ^2	Exp(*B*)	95% CI
Objective thirst	-.33 (.16)	4.24	.72	.52 - .98

Subjective thirst between conditions

Table 5

Logistic regression analysis of subjective thirst predicting future preference choice in the experimental condition

Predictor	*B* (SE)	*Wald* χ^2	Exp(*B*)	95% CI
Subjective thirst	-1.07 (.41)	6.73	.34	.15 - .77

Table 6

Logistic regression analysis of subjective thirst predicting future preference choice in the control condition

Predictor	*B* (SE)	*Wald* χ^2	Exp(*B*)	95% CI
Subjective thirst	-1.30 (.39)	11.22	.27	.13 - .58

Explanations

Table 7

Logistic regression analysis of explanations predicting future preference choice across conditions

Predictor	*B* (SE)	*Wald* χ^2	Exp(*B*)	95% CI
Explanations	-2.56 (.48)	29.09	.08	.03 - .20

Table 8

Logistic regression analysis of explanations predicting future preference choice in the experimental condition

Predictor	*B* (SE)	*Wald* χ^2	Exp(*B*)	95% CI
Explanations	-2.53 (.62)	16.40	.08	.02 - .27

Table 9

Logistic regression analysis of explanations predicting future preference choice in the control condition

Predictor	*B* (SE)	*Wald* χ^2	Exp(*B*)	95% CI
Explanations	-2.76 (.78)	12.59	.06	.01 - .29
