# Supplementary material for: “These pretzels are making me thirsty” so I’ll have water tomorrow: A partial replication and extension of adults’ induced-state episodic foresight
Source: PLoS One. 2021 Nov 17;16(11):e0259424. doi: 10.1371/journal.pone.0259424 (PMC8598010; doi:10.1371/journal.pone.0259424)
Supplement: S2 Appendix — (PDF) [file pone.0259424.s002.pdf]

S2 Appendix: Study 2 Consent Form

Consent Form (identifying information in this form has been removed for peer review)

Date: 2018-2019
Project Title: *Adults' Preferences*
Principal Investigator (PI): X

INVITATION
You are invited to participate in a study that involves research. The purpose of this study is to examine adults' preferences for the future (i.e., for tomorrow). This is an optional after-class activity that is not associated with or has no bearing on class performance.

WHAT'S INVOLVED
As a participant, you will be asked to fill out a demographics form. You will also be asked to make choices about your preferences for the future. The session will take approximately 5 minutes to complete and will take place in a lecture room at X.

POTENTIAL BENEFITS AND RISKS
This research will improve our knowledge about adult's future thinking. There are no known or anticipated risks associated with participation in this study.

CONFIDENTIALITY
All information you provide is considered confidential; your name will not be included with your data. The measures you complete will not be seen by anyone outside of the research team. Furthermore, because our interest is in the average responses of the entire group of participants, you will not be identified individually in any way in written reports of this research.

Data collected during this study will be stored in the laboratory of X, which will be secured at all times. Data will be kept for five years following publication in an academic journal, after which time all data will be destroyed by shredding paper records, or by deleting electronic records. Access to this data will be restricted to X, X, a graduate student, and research assistants in X laboratory (all of whom will agree to maintain confidentiality).

VOLUNTARY PARTICIPATION
Participation in this study is voluntary and participants have the right to request the withdrawal of their data. If you wish, you may decline to answer any questions or participate in any component of the study. If you choose to withdraw and data has been collected from you, the data will be destroyed. However, once you hand-in your questionnaire package to our researcher, we will be unable to remove the data that has been collected from you since we separate your name from the data you provide immediately and we cannot link the responses you provide back to your name.

PUBLICATION OF RESULTS

Results of this study may be published in professional journals and presented at conferences. Feedback about this study will be available as soon as data collection is complete in approximately one year. If you are interested in receiving feedback about the results of the study via email, please indicate below and X will send you a copy of the findings.

CONTACT INFORMATION AND ETHICS CLEARANCE

If you have any questions about this study or require further information, please contact X using the contact information provided above. This study has been reviewed and received ethics clearance through the Research Ethics Board at X **[File X-X-X]**. If you have any comments or concerns about your rights as a research participant, please contact the Research Ethics Office at X

Thank you for your assistance in this project. Please sign this form and return to the researcher.

CONSENT FORM

I agree to participate in this study described above. I have made this decision based on the information I have read in the Information-Consent Letter. I have had the opportunity to receive any additional details I wanted about the study and understand that I may ask questions in the future. I understand that I may withdraw this consent at any time.

Your Name: ____

Your Signature: ___________________________ Date: ____________________

If you are interested in receiving feedback about the overall results of the study, please provide your email address below.

Email Address: ____
